# Potential Value of TNF-α (–376 G/A) Polymorphism and Cystatin C (CysC) in the Diagnosis of Sepsis Associated Acute Kidney Injury (S-AK I) and Prediction of Mortality in Critically Ill patients

**DOI:** 10.3389/fmolb.2021.751299

**Published:** 2021-10-06

**Authors:** Hiba S Al-Amodi, Shimaa Abdelsattar, Zeinab A. Kasemy, Hanan M. Bedair, Hany S. Elbarbary, Hala F. M. Kamel

**Affiliations:** ^1^ Biochemistry Department, Faculty of Medicine, Umm Al-Qura University, Makkah, Saudi Arabia; ^2^ Clinical Biochemistry and Molecular Diagnostics Department, National Liver Institute, Menoufia University, Shebine Elkoum, Egypt; ^3^ Department of Public Health and Community Medicine, Faculty of Medicine, Menoufia University, Shebine Elkoum, Egypt; ^4^ Clinical Pathology Department, National Liver Institute, Menoufia University, Shebine Elkoum, Egypt; ^5^ Department of Internal Medicine, Renal Unit, Faculty of Medicine, Menoufia University, Shebine Elkoum, Egypt; ^6^ Department of Internal Medicine, Renal Unit, Faculty of Medicine, King Faisal University, Al-Ahsa, Saudi Arabia; ^7^ Medical Biochemistry and Molecular Biology Department, Faculty of Medicine, Ain Shams University, Cairo, Egypt

**Keywords:** sepsis associated acute kidney injury, TNF-α, polymorphism, cystatin C, critically ill patients

## Abstract

Sepsis Associated Kidney Injury represents a major health concern as it is frequently associated with increased risk of mortality and morbidity. We aimed to evaluate the potential value of TNF-α (−376 G/A) and cystatin C in the diagnosis of S-AKI and prediction of mortality in critically ill patients. This study included 200 critically ill patients and 200 healthy controls. Patients were categorized into 116 with acute septic shock and 84 with sepsis, from which 142 (71%) developed S-AKI. Genotyping of TNF-α (−376 G/A) was performed by RT-PCR and serum CysC was assessed by Enzyme Linked Immunosorbent Assay. Our results showed a highly significant difference in the genotype frequencies of TNF-α (−376 G/A) SNP between S-AKI and non-AKI patients (*p* < 0.001). Additionally, sCysC levels were significantly higher in the S-AKI group (*p* = 0.011). The combination of both sCysC and TNF-α (−376 G/A) together had a better diagnostic ability for S-AKI than sCysC alone (AUC = 0.610, 0.838, respectively). Both GA and AA genotypes were independent predictors of S-AKI (*p*= < 0.001, *p* = 0.002 respectively). Additionally, sCysC was significantly associated with the risk of S-AKI development (Odds Ratio = 1.111). Both genotypes and sCysC were significant predictors of non-survival (*p* < 0.001), suggesting their potential role in the diagnosis of S-AKI and prediction of mortality.

## Introduction

Acute Kidney Injury (AKI) is one of the most common complications among septic patients in the intensive care unit (ICU) and is frequently associated with a higher risk of morbidity and mortality. (Uchino et al., 2005). Significantly, both sepsis and AKI might increase host susceptibility for each other (Peerapornratana et al., 2019). Sepsis is one of the most common predisposing causes of AKI, and AKI is one of the earliest manifestations of sepsis in a complex pathophysiological manner. Therefore, the simultaneous presence of sepsis and AKI might be described as Sepsis Associated Kidney Injury (S-AKI) (Bellomo et al., 2017), which has been recently defined as a rapid worsening of renal functions with the existence of sepsis, with no other reasonable explanation of etiology (Manrique-Caballero et al., 2021), considering that most of the septic patients developed AKI earlier than being diagnosed (Rosen and Samuel, 2001). The pathogenesis of S-AKI has been the subject of extensive research focus, however, it is still inconclusively understood, apart from the concomitant relationship between dysregulated inflammatory and immunological response together with circulatory and tubular dysfunction (Manrique-Caballero et al., 2021).

Serum Creatinine (sCr) has been used as a gold standard for diagnosis of AKI, despite being affected by several biological factors such as age, sex, muscle build, and immobilization (Nejat et al., 2010a). Moreover, sCr might be raised late, until after the glomerular filtration rate (GFR) has declined to more than half (Bagshaw and Gibney, 2008). Usually, the interval between onset of kidney injury and consequent detectable increase in sCr may extend to 48 h (Herget-Rosenthal et al., 2004a; Nejat et al., 2010a). The occurrence of AKI will worsen with the prognosis and morbidity of sepsis, so early detection and management of S-AKI could improve patient outcomes. Cystatin C (CysC) has been the subject of extensive focus as a potential biomarker for the early diagnosis and prediction of AKI or as an alternate to the gold standard “creatinine” (Lagos-Arevalo et al., 2015). It is a member of the cys¬tatin superfamily of protease inhibitors and is secreted via nucleated cells. CysC is eliminated by the glomerulus filtration process then completely reabsorbed by renal tubules and later it is catabolized (Leem et al., 2017). In contrast to sCr, its levels are not considerably influenced by biological factors (Knight et al., 2004).

The role of genetic polymorphisms in pathological settings has recently been the subject of research studies, particularly for biomolecules that are involved in inflammatory and immunological responses (Fatani et al., 2018). Polymorphisms involving the promoter area of the Tumor Necrotizing Factor-Alpha (TNF-α) gene could relevantly modulate promoter function and affect its transcriptional activity, thus modifying the expression levels of TNF-α in immune and/or pathological settings (Jaber et al., 2004a), which would be linked to worst outcome (Mira et al., 1999) and mortality of patients with S-AKI (Jaber et al., 2004b). As a prominent mediator of the immunological and inflammatory responses, TNF-α is considered a key molecule for the interplay of S-AKI and a potential target in therapeutic approaches (Swaminathan et al., 2015). Given the relevance of the systemic inflammatory process in the pathogenic mechanism of AKI, the polymorphisms of inflammation-linked genes such as TNF-α might influence the susceptibility of an individual to AKI (He et al., 2018). The polymorphisms at the promoter region of TNF-α might alter the levels of proinflammatory mediators and cytokine response to stressful stimulation encountered at AKI development and progression, thus variants located at the promoter region could determine the severity of AKI, worst outcome, morbidity, and mortality (Susantitaphong et al., 2013).

Despite the TNF-α promoter, polymorphisms have been extensively studied (Bayley et al., 2004), but its role in S-AKI has not yet been fully elucidated. TNF-α-367 SNP was infrequently studied compared to other SNPs located in the promoter region of the TNF gene, and its role in critically ill patients and S-AKI is considered a matter of debat, especially when combined with serum biomarkers such as CysC.

In recent years, numerous biomarkers have been proposed for the diagnosis of AKI, most of which are in development or validation stages (Wettersten and Weiss, 2013; Darshi et al., 2016; Zhang et al., 2020). Nevertheless, it is still unclear, whether the diagnosis of AKI requires a single biomarker approach or multiple biomarkers approaches, taking into consideration the complexity and multifactorial aspects of AKI (Baker, 2005; Abdelsattar et al., 2021). Therefore, this study aimed to assess the value of sCysC and genetic determinant as TNF-α-367 SNP together for the diagnosis of S-AKI and prediction of adverse outcomes in critically ill patients.

## Materials and Methods

### Study Population

The current study was carried on a total of 400 subjects including 200 critically ill patients with no preexistence of AKI. They were admitted to the ICU of AL Noor Specialized Hospital Makkah in Saudi Arabia from July 2018 to August 2019. It included 200 apparently healthy control subjects, who were matched for age and gender to critically ill patients. This study was conducted according to the guidelines of the Declaration of Helsinki and was approved by the Institutional Review Board (IRB) of the Faculty of Medicine at Umm al-Qura University (IRB No. HAPO-02-K-012) and Ethical Committee at Al Noor Specialized Hospital (Approval No.4237). Informed consent was obtained from each participant in this study.

All included subjects were aged over 18 years old. Critically ill patients recruited in the current study were categorized into two groups: sepsis group (No. = 84; 42%) and acute septic shock group (No. = 116; 58%) according to the new consensus definition of sepsis and septic shock, in which sepsis was diagnosed by the existence of possible infection within the first 24 h of admission to ICU and confirmed by blood culture, in addition to consecutive development of organ dysfunction, which was characterized by a rise of total Sequential Organ Failure Assessment (SOFA) score of ≥2 points. Acute septic shock was diagnosed by the persistence of hypotension, requiring the use of vasopressors, and a rise of serum lactate levels to more than 2 mmol/L (Singer et al., 2016). Out of the 200 critically ill patients, a group of septic patients developed AKI (No. = 142; 71%) (S-AKI group), and the remaining did not (No. = 58; 29%) (non-AKI group). The S-AKI group fulfilled the diagnostic criteria of AKI in accordance with Kidney Disease: Improving Global Outcomes (KDIGO) clinical practice guidelines (Khwaja, 2012), which were recently updated to agree with the new definition of S-AKI (Manrique-Caballero et al., 2021).

Patients with any of the following criterion were excluded from the current study: patients aged less than 18 years, patients with an expected length of stay in ICU less than 24 h, patients who developed AKI owing to any other cause rather than sepsis, critically ill patients confirmed as having acute active hemorrhage, end stage renal disease, patients with malignant tumors, acute coronary syndrome, or acute pulmonary edema. Demographic and clinical data were composed of the patients’ medical records.

### Blood Sampling

Seven milliliters (ml) of venous blood was withdrawn from each patient under aseptic conditions and divided into two tubes. Then, 4 ml of whole blood was placed in two sterile vacutainer tubes containing ethylene diamine tetra acetic acid (EDTA). One of these tubes was stored immediately at −80°C until it was used for DNA extraction and SNP genotyping, the other EDTA tube was centrifuged at 1,500 rpm for 15 min, then the obtained plasma was used for immediate estimation of lactate. The remaining 3 ml was placed into a plain test tube, then centrifuged at 1,500 rpm for 15 min, the resultant serum was aliquoted into 2 eppendorf tubes which were further stored at −20°C until analysis. One was used for biochemical assessment of urea and creatinine and the other for CysC assessment by ELISA. Timing of blood sampling for serial assessment of serum urea and sCr was applied on the same day of admission (Day 0), then on Day 2 (after 48 h), and Day 4 (after 96 h) after ICU admission. Patients were followed up for 30 days as an endpoint of survival (Uhel et al., 2020).

### Biochemical Assessment

Assessment of serum urea and sCr was performed using an AU680 chemistry analyzer (Beckman Coulter/Olympus-NY, United States). Plasma lactate was performed using a Clinical Auto analyzer (Beckman Instruments, Fullerton, CA, United States). Serum cystatin-C was determined by the Enzyme Linked Immunosorbent Assay (ELISA) technique by a commercially available kit (Cat. No. DSCTC0; R&D Systems, Inc, Minneapolis, MN, United States). The assay is an enzymatically amplified one step sandwich-type immunoassay, which was performed in duplicate and according to the manufacturer’s instructions. The intra-assay and inter-assay coefficients of variation were < 10% and < 12%, respectively, corresponding to those reported by the manufacturer.

### Genotyping for TNF-α-367 (rs1800750) SNP

#### DNA Extraction

DNA extraction and Genotyping using Real-Time PCR technique were performed at the Molecular Biology Laboratories of Biochemistry Department, Faculty of Medicine, Umm AL-Qura University. Genomic DNA was extracted from all samples using Gene JET TM whole blood Genomic DNA purification Mini kit [Thermo Scientific EU/Lithuania] according to the manufacturer’s instructions. For the determination of purity of DNA, the DNA concentration of each sample was assessed using a Nanodrop spectrophotometer [UV spectrophotometer Q3000, Quawell Technology, Inc, United States].

#### Real-Time PCR (RT-PCR)

TNF α -376G/A (rs1800750) SNP was analyzed using real-time polymerase chain allelic discrimination technology, using TaqMan SNP genotyping assay kit [Thermo Fisher Scientific, Waltham, MA, United States] as previously described (Zambon et al., 2005)**.**

The targeted sequences of DNA were amplified using the specific primers for those sequences within the kit and catalyzed by DNA polymerase of TaqMan Master mix. Allelic discrimination was manifested by fluorescence signal emitted from the two TaqMan fluorogenic minor groove binder probes. The two TaqMan^®^ probes that targeted the TNF-α-367 (rs1800750) SNP site were as follows: one fluorescent dye detector that was perfectly matching the wild-type allele (allele G) and the other was the perfect match for the polymorphic allele (allele A).

The polymerase chain reaction assay was carried out starting with 5 μl of genomic DNA [1–10 ng], added to 10 μl of master mix, 3.75 μl of nuclease-free water, and 1.25 μl of the primer/probe mix. Thermocycling conditions were started by initial denaturation at 95°C for 10 min, then 40 cycles of denaturation at 95°C for 15 s, followed by 60°C of annealing for 1 min and finally 60°C of extension for 5 min. Primers were as follows; Forward [5′-CCCCTCCCAGTTCTAGTTCTATCTT-3´] and Reverse [5′-CCTATTGCCTCCATTTCTTTTGG-3´]. The probes used were FAM-CTGTCTGGAAATTAGAAG (376 A), VIC-CTGTCTGGAAGTTAGAAG (376 G) (Applied Biosystems, Foster City, CA, United States). Analysis of PCR data was conducted on Applied Biosystem 7500 RT-PCR ABI PRISM (Applied Biosystems, United States) software v.2.0.1.

### Sample Size

Based on past published research, reporting the difference regarding TNF-alpha –376 G/A polymorphism and genotypic frequency (AA) to be 9.2% between controls (2.6%) and total patients (11.8% distributed as 9.2% For sepsis and 15.2% for shock (Kothari et al., 2013), to achieve 90% power to detect this difference with a significance level of 5%, it was estimated that 195 subjects per group would be required. With a withdrawal/non-evaluable subject rate of 10%, a total of 216 per group subjects were recruited leading to a total of 200 participants in every group, with a response rate of 92.6%. According to Concato et al. and Peduzzi et al., the concept of a variable per 10 patients is acceptable for regression analysis (Peduzzi et al., 1996; Bujang and Baharum, 2016). The sample size was calculated at 11 variable, which is the maximum variable that could be used in this study. Using G Power 3.1, the sample size was calculated for regression analysis with power = (1-ᵦ) = 0.95 and CI 95% and it was estimated to be 178 patients. The total sample size was thus sufficient to study the primary outcome and regression analysis.

### Statistical Analysis

Data analysis was performed using IBM SPSS software statistical package version 20.0. (Armonk, SPSS Inc, Chicago, IL). The normality of distribution was tested by Kolmogorov-Smirnov test. For the two group comparisons as S-AKI and non-AKI, unpaired *t*-test was used for the comparison of parametric normally distributed data, along with Mann Whitney test for non-normally distributed non-parametric data, and Chi-squared test, Fisher’s exact test, or Monte Carlo tests for categorical variables. For the group comparison of sepsis, septic shock and controls, and ANOVA test were used, followed by Post Hoc test. Odds ratio (OR) was used to assess the risk at a 95% confidence interval. Sepsis, septic shock, and control groups were explored to find equilibrium with the Hardy-Weinberg equation. The receiver operating characteristic curve (ROC) was plotted to assess the diagnostic performance of the studied biomarkers and genetic determinants. The area under the curves (AUC) was calculated to assess the capability of studied markers to appropriately discriminate between patients with S-AKI and non-AKI. Based upon the ROC curve, the optimum diagnostic cut-off points for maximizing both the sensitivity and specificity for S-AKI diagnosis were studied. Logistic Regression analysis was accomplished to detect the most affecting factor for the prediction of S-AKI amongst the studied groups. Cox regression was used for assessing the relationship with overall survival. Kaplan-Meier Survival curve was also performed for the significant relationship with overall survival during a 30 days stay at ICU. The significance of the obtained results was considered at a value of 5%.

## Results

The current prospective study included 400 subjects (200 critically ill patients and 200 controls); their demographic, clinical, and biochemical data are presented in Table 1. The patient groups comprised 200 critically ill patients who were further subdivided into 116 with acute septic shock, of which 71 (61.2%) patients were male and 45 (38.8%) were female. Of the 84 patients with sepsis, 46 (54.8%) were male and 38 (45.2%) were female. The study cohort included 200 apparently healthy control individuals comprising 96 (48%) male and 104 (52%) female participants. The mean ± SD of age for septic shock, sepsis, and control subjects were 54.3 ± 13.1, 53.5 ± 7.4, and 52.3 ± 6.1 years respectively, all groups were comparable with a non-significant difference among them regarding age and gender (*p* > 0.05). Systolic and diastolic blood pressure were significantly higher in patients with sepsis than patients with septic shock (*p* < 0.001). Blood levels of lactate, first, second, and third estimation levels of urea and creatinine were significantly different among the studied groups (< 0.001). Serum levels of CysC showed highly significant difference among sepsis, septic shock, and control groups (*p* < 0.001), and the mean ± SD were 1.85 ± 0.48, 1.79 ± 0.62, and 0.73 ± 0.12 respectively. However, there was no statistical difference between sepsis group and septic shock group. During a 30 days stay at ICU, 73 (62.9%) patients in the septic shock group died and 53 (63.1%) patients in the sepsis group died with a non-significant difference between survivors and non-survivors in both groups (0.981) (Table 1).

**TABLE 1:** Demographic and clinical characteristics of studied groups.

	Controls (No. = 200)	Septic shock (no. = 116)	Sepsis (no. = 84)	*p* value
	Mean ± SD	Mean ± SD	Mean ± SD	
Gender	96 (48%)	71 (61.2%)	46 (54.8%)	0.073
Male No. (%)	104 (52%)	45 (38.8%)	38 (45.2%)	
Female No. (%)				
Age (years)	52.3 ± 6.1	54.3 ± 13.1	53.5 ± 7.4	0.153
SBP: Median (Range)	110 (100–125)	80.5 (66–95)	115 (95–211)	< 0.001^a^
DBP: Median (Range)	75 (70–85)	52 (40–65)	75 (59–118)	< 0.001^a^
sCystC	0.73 ± 0.12	1.79 ± 0.62	1.85 ± 0.48	< 0.001^a^
Lactate	1 ± 0.1	3.3 ± 1.3	1.4 ± 0.6	< 0.001^a^
Urea 1st	30.2 ± 3.5	70 ± 39.2	55.5 ± 35.9	< 0.001^a^
2nd	30.2 ± 3.5	109.4 ± 62.5	92.8 ± 43	< 0.001^a^
3rd	30.2 ± 3.5	136.4 ± 76.9	90.2 ± 34.1	< 0.001^a^
Creatinine 1st	0.9 ± 0.1	1.7 ± 1.8	1.1 ± 0.3	< 0.001^a^
2nd	0.9 ± 0.1	2.3 ± 1.6	1.6 ± 0.6	< 0.001^a^
3rd	0.9 ± 0.1	2.6 ± 1.9	1.7 ± 0.7	< 0.001^a^
Survival Survivors No. (%)	----	43 (37.1%)	31 (36.9%)	0.981
Non-survivors No. (%)	----	73 (62.9%)	53 (63.1%)	

a Significant. sCystC: serum Cystatin C. The statistics applied included Chi square test, ANOVA test for parametric data, and Kruskal-Wallis test for non-parametric ones.

The distribution of genotypes and allelic frequencies of the TNF-α gene (rs1800750) genotype are shown in Table 2. All identified genotypes were in equilibrium with Hardy-Weinberg. The genotype frequencies and allelic distribution of TNFα-376G/A (rs1800750) were significantly different among the studied groups (*p* < 0.001). The frequency of GG was 47.4% in the septic shock group, 64.3% in the sepsis group, and 83.5% in control subjects. A highly significant difference was observed in acute septic shock vs control and sepsis vs Control (*p* < 0.001). However, there was a borderline difference in the septic shock vs sepsis group (*p* = 0.051).

**TABLE 2:** Distribution of for TNF-α gene (rs1800750) genotypes among patient groups and controls.

	Controls (No. = 200)	Septic shock (No. = 116)	Sepsis (No. = 84)	*p* value	Sig. Bet. Groups		
	No. (%)	No. (%)	No. (%)		Septic shock vs sepsis	Septic vs control	Sepsis vs control
rs1800750							
GG	167 (83.5%)	55 (47.4%)	54 (64.3%)	< 0.001^a^	0.051	< 0.001^a^	0.001^a^
GA	29 (14.5%)	43 (37.1%)	23 (27.4%)	< 0.001^a^	0.018^a^	< 0.001^a^	< 0.001^a^
AA	4 (2%)	18 (15.5%)	7 (8.3%)				
GA + AA	33 (16.5%)	61 (52.6%)	30 (35.7%)				
Allele							
G	363 (90.8%)	153 (65.9%)	131 (78%)	< 0.001^a^	0.009^a^	< 0.001^a^	< 0.001^a^
A	37 (9.3%)	79 (34.1%)	37 (22%)				

a Significant.

Table 3 shows the highly significant difference of TNF-α -376 G/A (rs1800750) genotype frequencies between the S-AKI (No. = 142) subgroup and non-AKI subgroup (No. = 58). Among whole patients (*p* < 0.001) the frequencies of GG genotype in the total 200 patients with S-AKI were 36.6%, 45.8% for GA, and 17.6% for AA, respectively. In the non-AKI subgroup, genotype frequencies GG, GA, and AA were 98.3, 1.7, and 0% respectively. Meanwhile, sCysC levels were significantly higher in S-AKI compared to non-AKI in all patients (*p* = 0.011), and the septic shock group (*p* = 0.039), but non-significantly different in the sepsis group (*p* = 0.087).

**TABLE 3:** Distribution of TNF α gene (rs1800750) and Cyst C according to the S-AKI and non-AKI groups.

	Total patient (*n* = 200)			Septic group (*n* = 116)			Sepsis group (*n* = 84)		
	S-AKI (No. = 142)	Non-AKI (No. = 58)	*p* value	S-AKI (no. = 87)	Non-AKI (No. = 29)	*p* value	S-AKI (No. = 55)	Non-AKI (No. = 29)	*p* value
rs1800750	55 (38.7%)	54 (93.1%)	< 0.001	29 (33.3%)	26 (89.7%)	< 0.001*	26 (47.3%)	28 (96.6%)	*p* < 0.001^a^
GG									
GA	63 (44.4%)	3 (5.2%)		41 (47.1%)	2 (6.9%)		22 (40%)	1 (3.4%)	
AA	24 (16.9%)	1 (1.7%)		17 (19.5%)	1 (3.4%)		7 (12.7%)	0 (0%)	
CystC	1.9 ± 0.6	1.7 ± 0.4	0.011*	1.85 ± 0.66	1.62 ± 0.46	0.039*	1.91 ± 0.5	1.74 ± 0.41	0.087
Mean ± SD.									

a Significant MC: Monte Carlo *significant t: Unpaired *t*-test *p*: *p* value for comparing between AKI and non-AKI.

In the ROC curve analysis, the best cut-off point for sCysC was 1.93 ng/ml, and sCysC levels greater than 1.93 ng/ml demonstrated a sensitivity of 52.11% and specificity of 56.9% for S-AKI diagnosis among the whole patient cohort. It has recently been widely acknowledged that diagnostic accuracy can be improved substantially by combining multiple markers because a single biomarker does not attain enough sensitivity and specificity to be applied in clinical practice for decision-making. Combining two or more biomarkers, especially when including genetic markers, could help clinicians not only in diagnosis but also in choosing the most appropriate treatment, eventually improving in personalized management of the patient (Mazzara et al., 2017).

**FIGURE 1:**
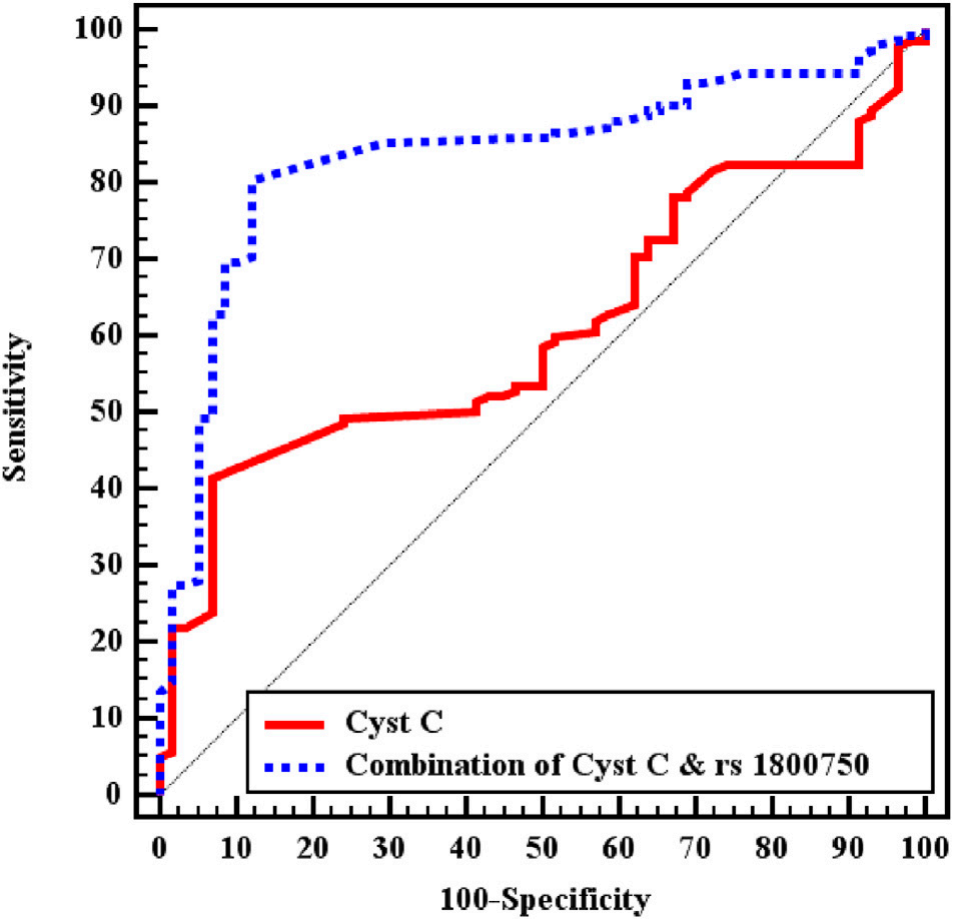
ROC curve analysis for CystC to discriminate S-AKI patients (No. = 142) from non-AKI (No. = 58) in total patients.

Combining sCysC and TNF−α −376 G/A had better discrimination ability with an increase in AUC from 0.610 to 0.838. Furthermore, the combination increased sensitivity and specificity to 85.21 and 70.69% respectively (Figure 1). Similarly, in the sepsis group, combining CysC and TNF−α−376 G/A instead of sCysC alone increased AUC from 0.676 to 0.843. Nevertheless, combining CysC and TNF−α−376 G/A signified the diagnostic performance for S-AKI in the septic group (*p* < 0.001) and increased AUC from 0.606 to 0.827 (Table 4).

**TABLE 4:** Validity (AUC, sensitivity, specificity) for Cyst C to discriminate S-AKI patients (No. = 142) from non-AKI (No. = 58) in all patients.

Studied group	Parameter	AUC	95% C. I	Cut off	Sensitivity%	Specificity%	PPV%	NPV%
All patients	Cyst C	0.610	0.532–0.689	≥1.93	52.1	56.9	74.7	32.7
	Combination of Cyst C and rs1800750	0.838	0.778–0.898	—	85.2	70.7	87.7	66.1
Sepsis group	Cyst C	0.676	0.561–0.791	≥1.18	92.7	34.5	72.9	71.4
	Combination of Cyst C and rs1800750	0.843	0.758–0.928	—	83.6	62.1	80.7	66.7
Septic shock group	Cyst C	0.606	0.088–0.715	—	—	—	—	—
	Combination of Cyst C and rs1800750	0.827	0.735–0.919	—	86.2	79.3	92.6	65.7

AUC: Area Under a Curve, CI: Confidence Intervals, NPV: Negative predictive value, PPV: Positive predictive value.

Logistic regression was performed in order to determine the risk for S-AKI occurrence on the probability that patients have−376 G/A (rs1800750) TNF-αgene mutation by univariant and multivariant regression in all 200 patients (Table 5). The heterozygote (GA) and homozygote (AA) are independent predictors for the occurrence of S-AKI, *p*= <0.001, *p* = 0.002 respectively. Systolic blood pressure, creatinine third estimation levels, and coexisting ischemic heart disease were accepted by the final model and were independent risk factors for S-AKI development, (*p* values = 0.018, 0.013, and 0.007 respectively). Odds ratio and 95% CI were 1.031 (1.005–1.057), 1.631 (1.110–2.398), and 0.064 (0.009–0.474), respectively. Serum levels of CysC were associated with the risk for development of S-AKI by univariate analysis only, *p* = 0.026, OR = 1.111 and 95% CI was (1.013–1.219).

**TABLE 5:** Univariate and multivariate Logistic regression analysis for S-AKI for different parameters in total patients (No. = 200).

	Univariate		Multivariate	
	*p* Value	OR (95%C. I)	*p* Value	OR (95%C. I)
SBP	0.006*	1.02 (1.01–1.03)	0.018*	1.03 (1.01–1.06)
DBP	0.764	0.99 (0.98–1.01)		
Cyst C	0.026*	1.11 (1.01–1.21)	0.139	1.12 (0.97–1.29)
Lactate	0.678	0.95 (0.77–1.18)		
Urea	0.006*	1.0 (1.0–1.02)	0.163	1.01 (0.99–1.03)
Creatinine 3rd	0.038*	1.28 (1.01–1.62)	0.013*	1.631 (1.11–2.39)
rs 1800 750 GG^®^		1.0		
GA	< 0.001*	20.61 (6.10–69.66)	< 0.001*	16.64 (4.16–66.48)
AA	0.002*	23.56 (3.078–180.371)	0.002*	65.88 (4.68–926.39)
Comorbidity				
DM	0.005*	2.86 (1.37–5.98)	0.617	0.73 (0.22–2.43)
HTN	0.166	0.62 (0.31–1.21)		
IHD	< 0.001*	0.05 (0.01–0.25)	0.007*	0.06 (0.01–0.47)

^®^: reference group OR: Odds ratio C.I: Confidence interval *: significant.

**FIGURE 2:**
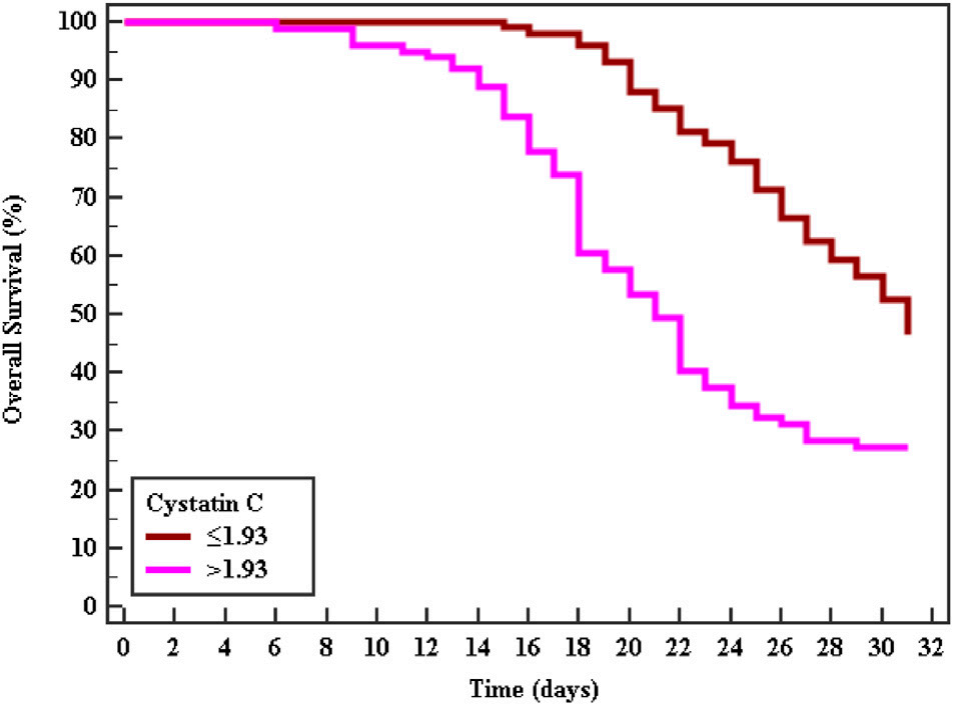
Kaplan-Meier survival curve for Overall Survival with CystC in total patients (No. = 200).

**FIGURE 3:**
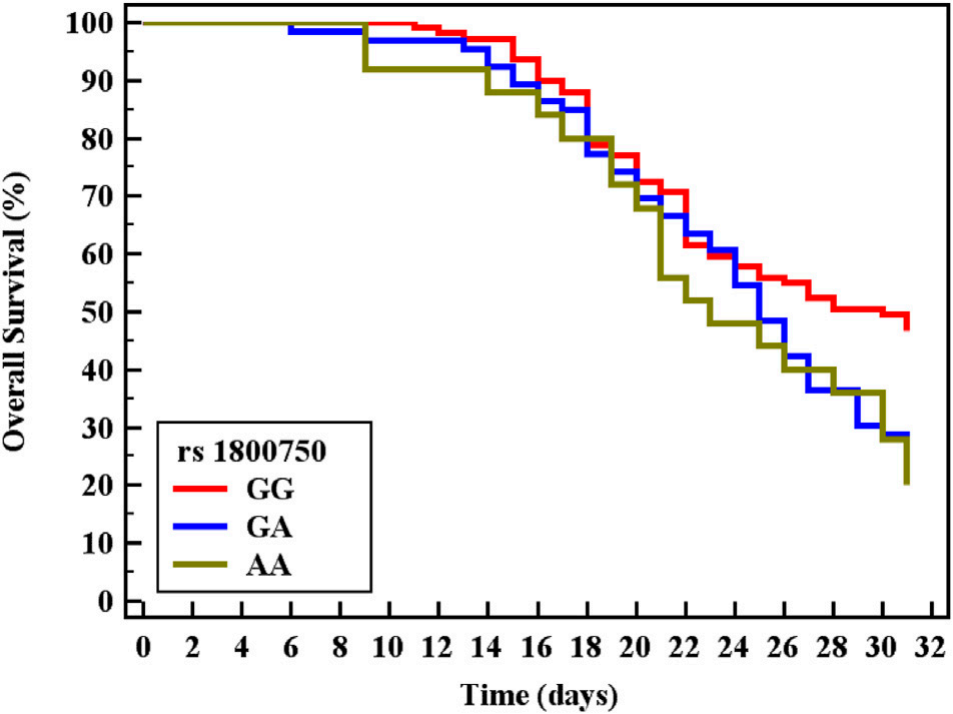
Kaplan-Meier survival curve for Overall Survival with TNF-α (rs1800750) in total patients (No. = 200).

Cox regression was performed to ascertain the relations of all variables with the overall survival of all patients (Table 6). Both of GA and AA genotypes of TNF−α −376 G/A (rs1800750) SNP, serial third estimation levels of urea and sCr, comorbidity of DM, and chronic kidney disease were predictors of non-survival by univariant analysis (*p* < 0.05). Serum levels of CysC were a strong and an independent predictor of mortality (*p* < 0.001) *via* univariate and multivariant analysis, HR, and 95% C.I were 1.154 (1.092–1.219) and 1.144 (1.077–1.216), respectively. As expected, AKI and coexisting ischemic heart diseases were also significant predictors of mortality by univariate and multivariate analysis (*p* < 0.001). The Kaplan-Meier Survival curve in Figures 2, 3 illustrate CysC levels at a cut-off of 1.93 ng/ml or greater. The GA and AA genotypes of TNF-α -376 G/A (rs1800750) SNP were significant predictors of non-survival in all patients (*p* < 0.001).

**TABLE 6:** Univariate and multivariate COX regression analysis for overall survival for different parameters in all patients (No. = 200).

	Univariate		Multivariate	
	*p* Value	HR (95%C. I)	*p* Value	HR (95%C. I)
Cyst C	< 0.001*	1.15 (1.09–1.21)	< 0.001*	1.14 (1.07–1.21)
Lactate	0.289	0.92 (0.81–1.06)		
Urea 3rd	< 0.001*	1.0 (1.0–1.01)	0.566	1.0 (0.99–1.01)
Creatinine 3rd	< 0.001*	1.24 (1.12–1.36)	0.887	1.01 (0.82–1.24)
Rs1800750		1.0		
GG^®^				
GA	0.032*	1.52 (1.03–2.23)	0.113	0.71 (0.47–1.08)
AA	0.026*	1.77 (1.07–2.96)	0.982	0.99 (0.55–1.78)
Comorbidity	< 0.001*	2.31 (1.61–3.30)	0.301	1.25 (0.81–1.94)
DM				
HTN	0.786	0.94 (0.63–1.41)		
IHD	0.007*	0.14 (0.03–0.58)	0.029*	0.19 (0.04–0.84)
CKD	0.006*	2.96 (1.37–6.39)	0.384	1.47 (0.61–3.50)
AKI	< 0.001*	4.27 (2.52–7.23)	< 0.001*	3.46 (1.88–6.34)

^®^: reference group OR: Odds ratio C.I: Confidence interval *: significant.

## Discussion

Sepsis is considered a frequent clinical challenge in critically ill patients as it is one of the most common participating factors of AKI (Shum et al., 2016). Moreover, it has been reported that approximately 60% of septic shock patients developed AKI with higher mortality rates (Plataki et al., 2011). Numerous studies have evaluated the probability that the genetic variability of cytokines could lead to alterations of the immune responses, with the occurrence of AKI in sepsis and septic patients (Phumeetham et al., 2012), thus indicating the role of TNF-α in creating and/or promoting the inflammatory reaction in critically ill patients (Winkelman, 2007; Zhang et al., 2017). Earlier prediction of S-AKI by genetic markers represents an important era in the application of personalized medicine, and might suggest novel targets for therapy in the future.

Our data showed a highly significant difference regarding genotype and allelic distribution of TNF−α (–376 G/A) rs1800750 among all the studied groups: acute septic shock, sepsis, and control subjects (*p* < 0.001). This result was in agreement with Kothari et al. who found a significant difference between patients with sepsis and septic shock which compares to the control group for the same investigated SNP in our study (rs1800750) (Kothari et al., 2013). Similarly, other SNPs such as TNF−α (–308, −238, +489) showed a significant difference between sepsis and acute septic shock patients (Gordon et al., 2004; Teuffel et al., 2010; Paskulin et al., 2011; Kothari et al., 2013). However, another opposing report did not find any significant difference in genotype and allelic distribution of TNF−α (–376 G/A) among septic patients (Georgescu et al., 2020). In the current study, a highly significant difference was observed between S-AKI and non-AKI groups for the genotype distribution of TNF-α (rs1800750) among whole patients, sepsis, and acute septic shock groups. In addition, GA and AA genotypes were independent predictors for AKI by both univariant and multivariant logistic regression analysis. SNPs located at the promoter area of TNF-α showed inconsistent associations with TNF-α synthesis and immunological response to various inflammatory or pathological processes, which indicate that the variability of genotype distribution at the promoter region might be the cause of the uniqueness of TNF response in individuals (Pujhari et al., 2012).

Numerous studies have recently explored the possible implication of TNF-α gene polymorphism at the promoter region and in pathological settings, however, only a few have found a significant association between the risk for development of S-AKI and the genotype distribution of the various SNPs (Bayley et al., 2004). A significant correlation has been encountered between the development of severe sepsis and the genotypes of the same investigated SNP in this study, TNF-α (–376 G/A), but the risk for AKI was the focus (Kothari et al., 2013). Disagreeing with our results, Cardinal-Fernandez and his group reported that TNF-α (–376 G/A) was not associated with AKI (Cardinal-Fernández et al., 2013). TNF-α (–308 G/A) SNP is closely located just beside TNF-α (–376 G/A) on the TNF promoter and was extensively studied (Verweij, 1999). Hashad and colleagues reported that the heterozygous and minor homozygous genotypes (G/A and A/A) of TNF-α (–308 G/A) SNP were significantly different in AKI and non-AKI patients and both genotypes were an independent risk for AKI development in patients with severe sepsis (Hashad et al., 2017). Similar to other reports, a significant association has been shown between the minor allele (A) of TNF-α (–308 G/A) and the severity of AKI (Susantitaphong et al., 2013), and organ dysfunctions in septic patients (Wang et al., 2017). In contrast, a larger study did not find any association between the above SNPs with AKI (Westphal et al., 2019). Likewise, TNF-α (–308) polymorphism was not associated with progression into AKI in patients with severe sepsis (Payen, 2019), and in patients following cardiac surgical interventions (Boehm et al., 2014). This discrepancy with our findings might be because our exact candidate SNP was rarely investigated previously in the same setting of sepsis, acute septic shock, or S-AKI. Moreover, the current study was focused only on one SNP, where other previous studies explored more than one SNP or haplotypes along the promoter region of the TNF-α gene. Another explanation is the inclusion of a healthy control group in our study, and on the other hand, some studies investigated the genetic distribution among patient groups only, the discrepancy of sample size, or ethnic groups.

Existing methods for the assessment of renal functions as sCr might be unable to detect very early changes, particularly in some patients with early tubular necrosis at presentation to ICU, (van Doorn et al., 2008). Because of its limited sensitivity and specificity in certain circumstances, CysC has been extensively studied as an alternate biomarker (Herget-Rosenthal et al., 2004b). In the current study, sCysC levels were significantly higher among sepsis and acute septic shock patients in comparison to the control group, and its levels were higher in S-AKI than non-AKI patients.

On ROC curve analysis of the studied markers for diagnosis of S-AKI, Serum CysC could significantly discriminate patients of S-AKI from non-AKI among whole patients and patients with sepsis, *p* values were 0.015 and 0.008 respectively. However, combining sCysC and TNF-α-367 SNP had better diagnostic performance than sCysC alone (*p* < 0.001). This combination increases AUC from 0.610 with sCysC alone (at cut off >1.93 mg/L) into 0.838 with both markers in the whole cohort of critically ill patients. AUC increased in the sepsis group, and in acute septic shock from 0.676 into 0.843 and from 0.606 into 0.827, respectively. Interestingly, the cut-off value of sCysC (1.18 mg/L) in the sepsis group was lower than in the whole patient group regarding discrimination between S-AKI from non-AKI. Likewise, combining sCysC with TNF-α-367 SNP had better diagnostic performance than sCysC alone, whereas specificity increased from 34.5 to 62.1% and positive predictive value increased from 72.9 to 80.7%. By linear regression analysis, sCysC levels were predictors of the occurrence of S-AKI (*p* = 0.026).

Several studies were in agreement with our findings that CysC is useful for diagnosis (Murty et al., 2013; Deng et al., 2017) and prediction (Herget-Rosenthal et al., 2004b; Åhlström et al., 2004) of AKI in critically ill patients in variable age groups, whether associated with sepsis or with another clinical setting (Che et al., 2019), regardless of the underlying precipitating factors. In the same context, a multicenter comparative study of several biomarkers reported that sCysC was the best diagnostic biomarker of AKI in comparison to both urinary N-acetyl-β-D-glucosaminidase and albumin/creatinine ratio, as AUC increased with the progression and severity of AKI (AUC for AKI and severe septic AKI was 0.738 and 0.839). Interestingly, the calculated AUC increased to 0.756 and 0.863, respectively by combining sCysC with urinary N-acetyl-β-D-glucosaminidase (which was very close to our calculated AUC), indicating the better performance of the combined panel of CysC and another biomarker for AKI diagnoses and prediction of its severity (Deng et al., 2017). Similarly, Murty and colleagues reported that sCysC was a better assessor of renal functions than sCr for early detection of AKI, even reflecting the declining of GFR upon progression and worsening of AKI (Murty et al., 2013). Likewise, another report revealed that serial daily estimation of sCysC in the first 3 days after admission to ICU was reflected in the progressive increase of AUC and diagnostic accuracy of acute renal failure in critically ill patients (Åhlström et al., 2004).

Contradicting with all the above findings, Mazul-Sunko and colleagues suggested that CysC might be of low diagnostic and prognostic value for AKI. However, the sample size was too small to be reliable (Mazul-Sunko et al., 2004). In a meta-analysis of nineteen studies that recruited more than three thousand patients, the range of sCysC cut-offs was 0.8–2.04 mg/L, the estimated pooled AUC was 0.96, sensitivity was 84%, and specificity was 82% (Zhang et al., 2011). These results were very close to those in our study. Nevertheless, the sensitivity, specificity, and cut off values for diagnosis of AKI range widely in various studies, which might be due to variability of age, clinical setting, precipitating factors, or the underlying causes of AKI in the studied cohort and the different assays used for the measurement of sCysC, as well as the exact timing of assessment of sCysC relevant to renal insult or ICU admission.

In the current study, sCysC was estimated at admission to ICU, which indicated its potential value for early detection and prediction of occurrence and/or progression of AKI. This observation is supported by others, who have reported that CysC was very valuable when it was estimated during the first 24 h from admission for early detection of AKI, whether assessed in serum (Soto et al., 2010; Kokkoris et al., 2012) or urine (Nejat et al., 2010b). In agreement with our findings regarding the predictive value of CysC, in a multi- ICU center study including 2,331 patients, sCysC was significantly higher in patients with AKI and was significantly associated with the risk of AKI development in patients with sepsis (Payen, 2019). Similarly, Safdar reported that sCysC was a good biomarker for diagnosing AKI in critically ill children (Zhang et al., 2011).

In our study, the estimated sCysC levels could significantly predict AKI on Day 0 of admission to ICU, while only the third estimated sCr (on Day 4) could predict the occurrence of AKI by univariate regression analysis. This finding is in line with another observational study in which sCysC predicted the occurrence of AKI earlier by one to 2 days than sCr (Herget-Rosenthal et al., 2004b). To the best of our knowledge, this study was the first to emphasize the potential value of combining the serum biomarkers of sCysC with genetic determinant TNF-α-367 SNP in the diagnosis and prediction of S-AKI in critically ill patients. Further identifying the various pathophysiological processes mediating AKI will eventually be essential for the development of target therapies and designing pharmacological trials (Okusa et al., 2016; Moledina et al., 2019).

Biomarkers as key players in translational medicine possessed great potential for the diagnosis of S-AKI. TNF-alpha (−376 G/A) and CysC have several advantages but might have disadvantages compared to the other widely used biomarkers in clinical practice, such as creatinine and other novel biomarkers that have been recently studied, such as Kidney injury molecule 1 (KIM-1) and neutrophil gelatinase-associated lipocalin (NGAL). The levels of sCysC rise earlier than sCr, reflecting early changes in renal function and the decline of GFR and thus might be an ideal and more precise index of GFR than sCr, with high sensitivity and specificity (Toffaletti, 2018). Even though serum CysC can diagnose AKI earlier than sCr (Herget-Rosenthal et al., 2004b), other reports have concluded that NGAL might have the advantage of being earlier than CysC in the diagnosis of AKI (Ling et al., 2008; Al-Beladi, 2015). The availability of automated immunoassay methods for assessment of CysC make it a more practical and clinically useful tool for the estimation of GFR in comparison to other biomarkers, meaning it might be used for routine investigations in the assessment of the kidney function of critically ill patients and could be of great help for the optimization of early detection, prevention, and treatment strategies for S-AKI(Finney et al., 2000). As a genetic biomarker TNF-alpha (−376 G/A) is not affected by biological factors such as age, gender, and muscle build, similarly CysC levels are not influenced by these factors. However, smoking, thyroid disorders, systemic inflammatory response, and corticosteroid therapy could significantly modify CysC levels (Knight et al., 2004). The serum levels of CysC might be paradoxically modified in patients with thyroid disorders and thus decreased in hypothyroid status and increased in hyperthyroid disorders (Jayagopal et al., 2003). Increased levels of serum CysC might be encountered in oncology patients with no evidence of underlying renal impairment, and levels were found to be higher in those patients, before and during chemotherapeutic cycles, compared to the reference population (Jones et al., 2017), thus oncological patients were excluded from this study. The effect of immunosuppressant therapy in renal transplant patients is reported to be dose-dependent, suggesting the induction of the promoter region of the CysC gene by corticosteroid, stimulating transcription and synthesis of CysC (Risch et al., 2001).

The Kaplan-Meier curve indicates that patients with minor alleles (genotype AA and GA) of TNF-α (–376 G/A) and patients with higher levels of sCysC had significantly shorter survival rates. Cystatin C was a strong independent predictor for mortality (*p* < 0.001) and additionally, S-AKI and the coexistence of ischemic heart diseases were also accepted by the Cox regression model as independent predictors of mortality (*p* < 0.001, 0.029 respectively). Critically ill patients and those with S-AKI were at high risk of death, especially those with coexisting morbidity and organ dysfunction (Lameire et al., 2013). This represents a strong incentive for researchers to investigate candidate biomarkers for the prediction of adverse outcomes. Recently, Helmersson-Karlqvist and colleagues supported our findings concerning sCysC. They concluded that a single assessment of CystC at admission to ICU was a strong predictor of mortality and even outstand sCr (Helmersson-Karlqvist et al., 2021). Contradicting our findings on the significant value of genotypes AA and GA of TNF-α (–376) in the prediction of mortality, most research did not find any association with their candidate SNPs and adverse outcomes or mortality (Gordon et al., 2004; Teuffel et al., 2010). This could be explained by the variability of statistical tests and power, the difference in ethnic populations, and different genotyping methods. Thus, the TNF promoter region might be one of the pivotal players in the progression and worst outcome of sepsis (Clark and Baudouin, 2006).

In conclusion, the current study suggested that a combination of serum CysC and both the heterozygous GA and homozygous AA of TNF-α (–376) genotypes have a better diagnostic ability for S-AKI than sCysC alone. The mutant genotypes were independent predictors of S-AKI and could significantly predict mortality. Moreover, sCysC was significantly associated with risk of S-AKI occurrence and an independent predictor of non-survival in critically ill patients. Our results highlight the potential value of TNF-α (–376) SNP and sCysC in the diagnosis of S-AKI and the prediction of worst outcomes, which might provide novel insight in the era of target therapy and offer additional therapeutic goals for the limitation of disease progression. Additionally, these findings might represent an advance in biomedical science in recognizing the various pathophysiological processes mediating the susceptibility of patients to S-AKI, which could have many implications for daily clinical practice.

The present study is subject to some limitations, as it was a one-center prospective study, and a larger number of patients was not available. We, therefore, recommend a wide-scale multicenter study that includes a larger number of patients to validate the current results. Moreover, a long-term follow up study for a possible association between response to specific treatment regimens and gene polymorphism would validate the use of personalized medicines in S-AKI. Additionally, we recommend further studies including other SNPs or haplotypes along with the TNF- α gene, examining the possible correlations between the various genotypes and their relative gene expressions and protein synthesis levels, to confirm possible relations with S-AKI pathogenesis and clinical outcomes.

## Data Availability

The datasets presented in this study can be found in online repositories. The names of the repository/repositories and accession number(s) can be found below: https://www.ncbi.nlm.nih.gov/nuccore/NG_007462
